# Health Implications of Climate Change: a Review of the Literature About the Perception of the Public and Health Professionals

**DOI:** 10.1007/s40572-018-0190-3

**Published:** 2018-02-08

**Authors:** Julia Hathaway, Edward W. Maibach

**Affiliations:** Center for Climate Change Communication, George Mason University, 4400 University Drive, MS 6A8, Fairfax, VA 22030 USA

**Keywords:** Climate change, Climatic change, Health, Public health, Perceptions, Attitudes, Opinions

## Abstract

**Purpose of Review:**

Through a systematic search of English language peer-reviewed studies, we assess how health professionals and the public, worldwide, perceive the health implications of climate change.

**Recent Findings:**

Among health professionals, perception that climate change is harming health appears to be high, although self-assessed knowledge is low, and perceived need to learn more is high. Among the public, few North Americans can list any health impacts of climate change, or who is at risk, but appear to view climate change as harmful to health. Among vulnerable publics in Asia and Africa, awareness of increasing health harms due to specific changing climatic conditions is high. Americans across the political and climate change opinion spectra appear receptive to information about the health aspects of climate change, although findings are mixed.

**Summary:**

Health professionals feel the need to learn more, and the public appears open to learning more, about the health consequences of climate change.

**Electronic supplementary material:**

The online version of this article (10.1007/s40572-018-0190-3) contains supplementary material, which is available to authorized users.

## Introduction

As stated in the introduction to the most recent US National Climate Assessment, “Climate change, once considered an issue for a distant future, has moved firmly into the present” [1]. The current impacts of climate change include increases in certain types of extreme weather (e.g., heat waves, heavy precipitation) and climatic events (droughts), increased water and food insecurity, rising sea levels and ocean acidification, damage to ecosystems and biodiversity, damage to societal infrastructures, and most directly germane to this article, a range of direct and indirect harms to human health [1–3].

The US Climate and Health Assessment identified seven major categories of health impacts from climate change, including increased morbidity and mortality from increasing extreme temperatures; temporary reductions in air quality from smog and smoke; increases in extreme weather and climatic events; increasing vector-borne diseases; increasing water-related illness; decreasing food safety, nutrition, and distribution; and mental health conditions including anxiety, depression, and substance use. While anyone’s health can be harmed by climate change, some people are at greatly increased risk including young children, pregnant women, older adults, people with chronic illnesses and disabilities, outdoor workers, and people with fewer resources [3]. Some of these health impacts are just now emerging, but others have been harming the public’s health for years [4].

Although much remains to be learned about the impacts of climate change on human health, the fact that climate change is a major public health threat has been observed by local communities around the world and has been well documented by climate and health experts [1–3, 5••, 6]. Indeed, climate change is seen by some experts as “the biggest global health threat of the 21st century” [7]. The extent to which the general public and practicing health professionals—including clinical and population health professionals—around the world are aware of the health relevance of climate change, however, is unclear. Therefore, we conducted a systematic literature review to answer three research questions focused on health professional and public understanding of the human health relevance of climate change:

1. How do health professionals perceive the health implications of climate change?
2. How does the public perceive the health implications of climate change?
3. How does the public react to information about the health implications of climate change?

Furthermore, we stratified the review into two linguistically defined geographies: English-speaking nations and non-English-speaking nations.

## Methods

We used an iterative search process to locate research for consideration and review. First, between February 8 and February 22, 2017, we searched Google Scholar utilizing search terms “climate change,” “climatic change,” “health,” “public health,” “perceptions,” “attitudes,” and “opinions.” The search period covered all publications available by March 2, 2017. Between February 22 and March 2, 2017, we searched Medline, Global Health, Psych Info, Web of Science, GreenFILE, and Communication and Mass Media Complete, using mesh terms “perceptions and attitudes” and “perceptions and attitudes and opinions” with “climate change” or “climatic change” and “health” or “public health” where possible. Concurrently, we requested citations from some authors who publish in this area and posted a similar request to a relevant listserv (International Environmental Communication Association).

With one exception (a nationally representative poll conducted by our research team that contained unique relevant information [8]), only English language, peer-reviewed studies were included in the review. Additional exclusion criteria were the following: research done on convenience samples (except in the case of in-depth interview studies, for which convenience samples are appropriate); inadequate information provided on survey response rate; and focus group research (due to concerns about validity of such data [9]).

Research questions were as follows:

RQ1: How do health professionals perceive the health impacts of climate change?

RQ2: How does the public perceive the health impacts of climate change?

RQ3: How does the public react to information about the health implications of climate change?

English-speaking countries included Australia, Canada, the UK, and the USA. One 24-nation study also included New Zealand [10].

A brief summary of each study identified was prepared; these are included in a Supplement for this article. From those summaries, the following results were synthesized.

## Results

RQ1: How do health professionals perceive the health impacts of climate change?

### English-Speaking Nations

We identified a total of 12 studies conducted with health professionals in English-speaking nations: seven with public health department personnel (six in the USA [11–16] and one in Canada [17]), four with physicians (three in the USA [18–20] and one in Australia [21]), and one with dieticians in the USA [22]. On the whole, a large majority of both clinical and population health professionals with whom research has been conducted understand that climate change is occurring, and that it is already harming or has the potential to cause harm to the public’s health in the near future.

In health departments, different groups of professionals—specifically directors, environmental health directors, and nursing directors—appear to have largely similar views of the public health relevance of climate change, but some differences are evident both within and between groups.

Most public health department directors see climate change a potentially serious public health problem in their jurisdiction, although this is not universally the case. In 2008, a majority (69%) of American local public health department directors perceived climate change to be occurring in their jurisdiction—whereas only 11% did not—while 78% felt their jurisdiction would experience climate change over the next 20 years [11]. However, a 2012 replication of the 2008 study found that polarization may have occurred, with most directors becoming more certain that climate change is already occurring in their jurisdiction (an increase of 9.3%), while other directors became more certain it is not happening in their jurisdiction (an increase of 11.3%); the proportion who were not sure either way had declined [16].

Similarly, by 2012, more directors had become more certain that over the next 20 years, their jurisdiction would experience one or more serious public health problems as a result of climate change (an increase of 17.8%), while more directors also became more certain their jurisdiction would not (an increase of 9.3%). Two state-based surveys of health department directors showed divergent profiles: in 2008, nearly all directors in California (94%) perceived climate change to be a threat to public health [12], whereas in 2009, only a minority of directors in New York State (32%) perceived climate change to be occurring yet in their jurisdiction [13].

Most environmental health directors nationwide feel that climate change will have serious health impacts globally (65%) and in the USA (56%); however, fewer than half (46%) believe their jurisdiction will experience serious impacts [14]. Similarly, nearly all public health nursing directors nationwide (90%) perceive that human-caused climate change is happening (90%), and most (65%) feel its health-related impacts will be a serious public health issue in the next 20 years [15].

The specific public health risks of greatest concern to public health personnel appear to be heat-related impacts, displacements, disruptions in health care due to storms and floods, vector-borne illness, air quality-related conditions, and mental health problems [11, 12, 14, 17]. Relatively few health departments have developed programs to address these problems, and most respondents indicated their department will need assistance (including expertise, additional people, and finances) to make significant progress [11–14, 16, 17].

Among physicians, a similar profile emerged. Most clinicians are convinced that climate change is happening and is already beginning to affect the health of some of their patients. Three physician surveys—of African-American physicians, thoracic specialists, and asthma and allergy specialists—all found that a sizable majority of respondents felt they had patients whose health was already being harmed by climate change in a variety of ways. The health impacts most commonly identified were air pollution-related increases in severity of chronic disease, such as asthma, COPD, and cardiovascular disease; allergic symptoms; heat-related effects; vector-borne illnesses; diarrheal diseases from food-/water-borne agents of infection; and injuries due to extreme weather [18–20].

Despite their experiences to date with health impacts of climate change, most physicians feel they lack knowledge about the topic, and large majorities support increased education on the health aspects of climate change in the form of continuing medical education, undergraduate medical education, patient education materials, and medical association policy statements. Large majorities also say that physicians and their associations should be involved in advocacy pertaining to the health effects of climate change [18–20].

### Non-English-Speaking Nations

Six surveys of health professionals’ perceptions of and knowledge about climate change have been done in non-English-speaking nations. They include Ethiopian health science students [23], Indian medical residents [24], Cambodian health professionals [25], Chinese hospital-based nurses [26], and Chinese public health professionals [27, 28]. In all cases, a large majority of respondents perceived that climate change is harmful to health, but their self-assessed knowledge was low, and their perceived need to learn more was high.

In addition, one study conducted in the European Economic Area, including all European Union countries as well as Norway, Iceland, and Lichtenstein, found that a large majority of national infectious disease experts agreed that climate change would affect vector-borne (86%), food-borne (70%), water-borne (68%), and rodent-borne (68%) diseases in their countries [29].

RQ2: How does the public perceive the health impacts of climate change?

### English-Speaking Nations

Ten studies have been done to assess public understanding of the health impacts of climate change in English-speaking countries; most of this research has been conducted in Canada [30–33] and the USA [8, 11, 31, 34–36]. Overall, it appears that relatively few Canadians and Americans associate climate change and health harms; most report they have given little thought to the issue [31, 37]. The depth of participants’ knowledge and risk perceptions is difficult to assess because people’s answers to open-ended questions give the impression of relatively little knowledge and risk perception [31, 34, 35, 38], whereas people’s answers to closed-ended questions demonstrate much higher level of risk perception [31, 34, 35, 39]. Specifically, only a minority of survey participants answer open-ended questions in ways that suggest they know anything about how climate change harms health, or who is most likely to be harmed [11, 38]. However, when asked specific closed-ended questions, a majority of respondents answer in a manner indicating that they do perceive climate change to be harmful to health [31, 37].

There are at least two possible explanations for the discrepancy between people’s answers to open-ended versus closed-ended answers about climate and health. One is that many people are more knowledgeable and perceive more risk than their open-ended responses suggest, but because the issue has low salience to them, specific health impacts and risk groups do not come to mind unless specifically prompted (by follow-on or closed-ended questions). Another possibility—made plausible by the fact that the majority of people surveyed hold negative views about global warming—is that many people do not have prior perceptions about the health relevance of climate change, but when asked closed-ended questions about the topic, they draw on their preexisting negative views about the issue in general to generate answers to specific questions about climate and health. Which explanation(s) best account for the discrepancy between the findings resulting from open-ended and closed-ended questions is not yet clear.

Another important finding is that in studies in the USA and UK, people have a strong tendency to see climate change as less threatening to their health and to their family’s health than to other people’s health—with escalating levels of health threat accorded to people in their community, to Americans in general, and to people worldwide [8, 36, 40].

The one clear exception to finding that few people can identify specific health harms from climate change comes from a study of Inuit elders and seniors in Nunatsiavut, Canada. The majority of Inuits interviewed felt that climate change is harming their health through reduced physical activity (due to a shorter ice season), compromised nutrition (due to reduced catch of wild game), and increased stress and substance use [32].

In summary, a majority of people in Canada (except possibly Inuit elders) and the USA appear to know relatively little about the health impacts of climate change, but many hold negative views of the health impacts of climate change regardless, or they appear willing to accept that climate change is harmful to human health.

### Non-English-Speaking Nations

Eight studies have assessed public understanding of the health harms associated with climate change in the non-English-speaking world. Three of these studies have been conducted in Africa (two in Tanzania and one in Nigeria [41–43]) and four in Asia (Bangladesh, Nepal, Tibet, and Vietnam) [43–46], with one additional study in Malta [31]. In all cases but one (Malta), this research has focused on especially vulnerable regions and populations.

Awareness of climate change per se tends to be low—with Hanoi, Vietnam serving as an exception (at approximately 75% [47])—but all of the studies found very high rates of perceived changes in climatic conditions that are consistent with climate change, including changes in precipitation and temperature. All of the studies also show very high rates of awareness of health harms due to these changing climatic conditions. These include increases in food insecurity (Tanzania, Nigeria, Bangladesh [42, 43, 48]) and water insecurity (Nigeria, Bangladesh [43, 48]); illness (Malta, Nigeria, Nepal, Vietnam [31, 46–48]) and stress (Nigeria [48]); injuries from extreme weather events (Bangladesh [43]); and heat risk (Tibet [45]).

RQ3: How does the public react to information about the health implications of climate change?

Eleven studies have assessed public reactions to information about the health implications of climate change [10, 49–58]. All but one was done in American populations. The exception, a study conducted in 24 nations [10], included the following nations in addition to the USA: Australia, Brazil, Chile, China, France, Germany, Ghana, Iceland, Israel, Japan, Mexico, Netherlands, New Zealand, Norway, Poland, Russia, South Africa, South Korea, Spain, Sweden, Switzerland, Venezuela, and the UK. No firm conclusions can be reached, but some tentative conclusions seem defensible.

Americans across the political and climate change opinion spectra appear receptive to information about the health aspects of climate change [49–51]. Other studies, however, suggest limitations. One study found a small beneficial effects of providing health-framed climate information, but also found that the effect was neutralized by climate change denial counter-messaging [51]. Another found no differential impact of a health-framed message [52], and a third found positive effects for self-identified Democrats, but negative effects (i.e., a boomerang effect) among self-identified Republicans [53]. Conversely, one small study with people who were particularly vulnerable to the health impacts of climate change (based on their health and SES status) found that participants responded favorably to simple information about climate and health, becoming more certain that climate change is happening and that it may affect their health, and they gained knowledge about who is most vulnerable to such impacts [54].

One study—which examined how likely various types of information about climate change are to be shared from person to person—found that information about the health impacts of climate change was shared with greater frequency than information about several other types of climate impacts [55].

A small field study of homeowners found that information about the environmental and public health externalities of electricity production, such as childhood asthma and cancer, was more effective at motivating home energy-saving behavior than information about saving money, especially among households with children [56].

A series of studies conducted by Levine and Kline [57], however, provides a cautionary note. In three experiments—field experiments and web-based experiments—they found that climate messages intended to make people concerned about their own health successfully heighten people’s concerns about climate change, but paradoxically they also reduce people’s rate of political participation (e.g., signing a petition, or joining a climate advocacy organization) to express their concern. The hypothesized mechanism of the paradoxical finding is that when people feel their material resources are threatened (in this case, their health), their response is to conserve their resources by avoiding additional commitments (presumably so as to better withstand resource constraints that may be forthcoming).

The only research conducted beyond the USA was a 24-nation study of university students that also included community samples in 10 of the nations. Participants were not presented with information about climate and health, but rather were asked to imagine what their country will look like in the future—in 2050—when people have taken action aimed at preventing climate change. They were then asked how much better, or worse, various conditions in their society would be at that time, including disease and pollution. Participants who believed that climate change action would reduce dysfunction in society, including realizing the health benefits of reducing disease, were significantly (albeit weakly) more likely to intend to perform climate change-relevant citizenship actions (e.g., contact an elected official) and household actions (e.g., install products to save energy) over the next 12 months, and to donate to an environmental organization [10].

## Conclusion

Given the rapidly mounting evidence of the many current significant health risks posed by climate change [1–3, 6], our review makes clear the relative paucity of assessment research aimed at illuminating health professional and public understanding of these risks; we found only 46 English language peer-reviewed studies that addressed any of the three basic research questions that we posed. Informing, educating, and empowering populations at risk, and the health professionals who serve them, are an essential function of public health, and performing this function effectively requires assessment of what these key audiences already know [59]. Developing effective climate change adaptation programs that protect the public’s health from increasingly extreme weather, climate variability, and other changing climatic conditions will require sustained professional and public education initiatives [60–64]. The far-reaching health effects of climate change have important implications for how nations build, organize, and manage health care systems [65], particularly in vulnerable communities and low-income countries where the worst effects will be felt.

Our conclusion from the sparse evidence reviewed is that many health professionals in North America and in some parts of Asia and Africa have at least basic awareness that climate change is a proximal and serious health threat to the public’s health, and they are acutely aware of both their need to learn more about the risks and how to manage them; they are also aware of the need for additional resources—in the form of expertise, personnel, and financing—to mount effective population health responses.

Our conclusion about public awareness—again, based on sparse evidence—is that most residents of developed countries know little or nothing about the health relevance of climate change, but are open to learning such information, and are inclined to believe that it may be harmful to health. Conversely, most vulnerable residents of developing nations do appear to recognize that specific climatic conditions where they live are changing, and those changing conditions are causing deleterious health impacts, although most have not ever heard the terms global warming or climate change.

The news media can play an important role in informing the public—and health professionals—about the health implications of climate change. Historically, however, the issue has received little press attention. The overall amount of climate change news reporting has waxed and waned considerably over the past several decades, with more reporting occurring around major scientific (e.g., IPCC) and political (e.g., the Paris Climate Agreement) events, but the total amount of climate reporting is a very small fraction of the total “news hole” (i.e., the total amount of news reported)—less than 1% worldwide [66]. In turn, climate and health stories are a small fraction of all climate news stories. In select American national and local newspapers, for example, the proportion of climate change stories that focus on climate and health stories has been shown to be in the range of 5 to 14% [67, 68]. Developing a sustained news media outreach strategy may provide a cost-effective basis for educating the public about climate change health risks and prevention options.

Finally, some health experts have called for efforts to heighten public engagement in limiting (i.e., mitigating) climate change by highlighting the health impacts of climate change [69] and the benefits of reducing greenhouse gas emissions [60, 70]. Our conclusion from the studies reviewed for RQ3 is that there may be merit to this communication strategy. At least some evidence suggests that learning about the health relevance of climate change and climate solutions helps stimulate public engagement, particularly among vulnerable people and households, and conservatives. The largest study to examine this question to date—which was not included in our review (but is currently under review)—found that American adults who read brief essays about the eight categories of health impacts of climate change—as summarized from the National Climate and Health Assessment [3]—became more cognitively and affectively engaged in the issue of global warming, especially people with a chronic illness, and political moderates and conservatives. These findings are promising in that the health angle can bring new constituencies and perspective to local, state, and national policy discourses about climate change mitigation [71]. This would appear to be a promising area for further research.

It is worth noting that accelerating the transition away from fossil fuels and toward clean renewable energy is arguably the most important global action that can be taken to limit climate change and prevent its health consequences [1, 3, 6]. American polling research finds high public support for this proposition and finds that the robust public support for clean energy is largely driven the perception that clean energy sources are healthful energy sources [72, 73].

## Electronic supplementary material

ESM 1 (DOCX 81 kb)
