# Supplementary material for: Health Implications of Climate Change: a Review of the Literature About the Perception of the Public and Health Professionals
Source: Curr Environ Health Rep. 2018 Feb 8;5(1):197–204. doi: 10.1007/s40572-018-0190-3 (PMC5876339; doi:10.1007/s40572-018-0190-3)
Supplement: Supplementary file 1 — (DOCX 81 kb) [file 40572_2018_190_MOESM1_ESM.docx]

Supplement

Health Implications of Climate Change: A Review of the Literature About the Perception of the Public and Health Professionals

**This file includes:**

Appendix A

**Appendix A. Brief Summaries of Each Study Reviewed, by Research Question**

**RQ1: How do health professionals perceive the health impacts of climate change?**

English-speaking nations

*Public Health Department Personnel*

In 2007, using a telephone survey of local public health department directors in the United States (n=133; randomly sampled; 61% response rate) the authors (1) found that a majority (69%) perceived climate change to be occurring in their jurisdiction – the most common impacts being related to heat waves (56%), storms and floods (47%), droughts and wildfires (47%), vector-borne illness (42%) and air pollution (42%) – with more (78%) perceiving their jurisdiction will experience climate change over the next 20 years. About half (51%) indicated climate change adaptation was a priority for their health department, but many were not yet implementing programs. Factors that may have influenced this discrepancy between problem recognition and programmatic responses included: most respondents felt personnel in their health department lacked knowledge about climate change; relatively few respondents felt their health department, their state’s health department, or the CDC had the necessary expertise to help them create an effective mitigation or adaptation plan; and most respondents felt that their health department needed additional funding, staff and training to respond effectively. The authors concluded: “these data make clear that climate change adaptation and prevention are not currently major activities at most health departments, and that most, if not all, local health departments will require assistance in making this transition.”

In 2007, in an online survey of all local public health officers in California(2) (n=34; 56% response rate) found that nearly all respondents (94%) perceived climate change to be a threat to public health, with majorities perceiving a range of specific serious risks to health including: extreme weather (91%), wildfire (86%), air pollution (79%), heat mortality (79%), vector-borne illness (76%), and water contamination (62%). Relatively few departments (about 25%) had developed programs to deal with health impacts of climate change – most commonly heat emergency plans and land-use plans (with other government agencies) – and most did not feel well equipped in terms of financial resources (93%), staff resources (79%) and information resources (71%) to cope with the threat.

In 2009, in an online survey of city and county health officials in New York State (n=22; census; 54% response rate) Carr and colleagues(3) found much lower rates of engagement than a parallel national survey conducted earlier(1). A minority of respondents (32%) perceived climate change to be occurring in their jurisdiction, while slightly more (39%) thought their jurisdiction would experience climate change over the next 20 years (39%). Only one quarter (25%) indicated climate change adaptation was a priority for their health department, and few (14%) felt their health department had ample expertise to assess the potential public health impacts of climate change in their jurisdiction. Many (48%) were not even sure if additional resources would significantly improve their department’s ability to deal with climate change; some open-ended comments suggested that departments felt challenged to achieve even the basic mandated public health functions, and climate change adaptation was not among them.

In 2010, in an online survey of environmental health directors in all local, state and territorial health agencies in the United States (n=225; census; 27% response rate) Syal and colleagues(4) found the majority felt that climate change would have serious health impacts globally (65%) and in the U.S. (56%), however, fewer (46%) believed their jurisdiction would experience serious impacts. The specific impacts seen to be of greatest concern for their jurisdiction were air quality-related illness (49%), vector-borne disease (47%) and flooding displacement (43%). About half (49%) felt their department had a responsibility to address climate change adaptation. Two-thirds (66%) felt their department lacked ability and preparedness to address the impacts. Commonly cited barriers to their ability to take adaptation actions included funding, staff training, and GIS software. Perceived climate change health risks were strongly associated with the number of climate change adaptation programs the environmental health department had established, while liberal political views of the director were strongly associated with perceived risk. The authors concluded: “highlighting the likelihood and severity of localized impacts may increase adoption of adaptation programming…”.

In 2010, in an in-depth interview study of 49 municipal health officials in Ontario, Canada (plus 4 provincial and federal health officials) Paterson and colleagues(5) found that health officials were concerned about how a changing climate could exacerbate existing health issues or create new health burdens, specifically extreme heat (71%), severe weather (68%) and poor air-quality (57%). As a result of local health department leadership, adaptation was taking the form of mainstreaming climate change into existing public health programs. Further progress was felt to require federal support, political will, and inter-agency efforts, as a lack of resources constrained the sustainability of long-term adaptation programs and the acquisition of data necessary to support effective policies.

In 2010, in an online census survey of nursing directors, American state and local health departments (n=176; 22% response rate), Polivka and colleagues(6) found that a large majority (90%) understood that human-caused climate change is happening. Approximately half or more correctly identified (from a list) that a range of conditions are increasing due to climate change, including vector-borne diseases, flooding related displacements, mental health conditions, air quality-related conditions, food borne diseases, disruption of health care services due to extreme weather, water-borne infectious disease and heat related illnesses; although out of 12 possible impacts, the mean selected was 5 (sd=4), and the modal response was zero. Most respondents (65%) felt that the health-related impacts of climate change would be serious in the next 20 years.

In 2012, using an online survey of directors of American local health departments (n=133; randomly sampled; response rate=61%), Roser-Renouf and colleagues(7) replicated an earlier survey of the same population(1) and found evidence of increasing polarization of views about climate change. More respondents in 2012 were certain that the threat of local climate change impacts either does or does not exist, and fewer were unsure; in 2012 approximately 10% said it is not a threat, compared to 1% in 2008. Adaptation capacity had decreased in several areas including: perceived departmental expertise in climate change risk assessment; departmental prioritization of adaptation; and the number of adaptation-related programs and services departments provided.

*Physicians*

In a paper & pencil survey of general practicing physicians in one rural district of New South Wales, Australia (n=68; response rate=27%), Purcell and McGirr(8) found that most respondents (76%) felt that climate change has substantial public health implications, and that farmers (87%), children (83%), homeless (81%), the elderly (80%), and native peoples (79%) were most vulnerable to the changing climate. A majority felt that climate change was likely to already be causing various health problems in their community – including vector-borne diseases (61%), water insecurity (58%), heat-related illness (57%), infectious diseases (55%), food insecurity (55%), and cardiorespiratory illnesses (51%) – while much larger majorities felt these impacts were likely to occur in the future (ranging from 69% to 82%). Over half the respondents felt that general practicing physicians should advocate to patients (65%), the local community (62%), and government (59%) on climate change and health.

In 2014, in a mixed modality (paper/pencil and email) survey of African-American physicians (n=284; response rate=30%), Sarfaty and colleagues(9) found that over 97% of respondents indicated that climate change is happening, 88% reported that it is relevant to direct patient care. Only a minority reported being “very” (6%) or “moderately” (18%) knowledgeable about the association between climate change and health impacts, however, with reporting being only “modestly” (48%) or “not at all” knowledgeable (28%). A majority of respondents indicated that, over the past decade, climate change had “harmed” people in their city or county “a great deal” (20%) or a “moderate amount” (46%), and that it had affected the health of *their own* patients “a great deal” (18%) or a “moderate amount” (43%). The most common health effects of climate change that respondents observed among their patients were injuries due to severe weather (88%), air pollution-related increases in severity of chronic disease (88%), increased allergic symptoms (80%), and heat related effects (75%). Other conditions affecting their patients included vector-borne infections (Lyme disease or West Nile Virus) (58%), diarrhea from food/waterborne agents of infection (56%), and mental health problems related to these health issues (40%). A large majority of respondents indicated that *certain groups* of people will be disproportionately affected by climate change, including people with chronic diseases (88%), people living near or below the poverty line (86%), young children ages 0–4 (83%), adults over age 60 (80%), and people of color (73%). A large majority of respondents agreed that each of three specific resources would be helpful to them: *“continuing medical education (CME) on climate change and health”* (41% strongly agree, 48% agree); *“patient education materials”* (41% strongly agree, 45% agree); and *“policy statements provided by my professional associations”* (34% strongly agree, 48% agree). The majority of survey respondents felt that physicians and medical societies have a role to play in addressing climate change and health, including education of doctors, patients and the public, as well as leadership and advocacy.

In 2014, in an email survey randomly selected U.S. members of the American Thoracic Society (n=915; response rate=17%), Sarfaty and colleagues(10) found that a large majority of respondents felt that climate change is happening (89%), and that it is driven by human activity (68%). A minority of respondents (38%) felt “moderately” or “very” knowledgeable about the association between climate change and health, while nearly half (44%) felt only “modestly” knowledgeable. Nearly all (87%) felt that climate change is relevant to direct patient care, and a majority think it is already affecting the health of their own patients in various ways including: air pollution–related increases in severity of chronic disease (77%), increased allergic symptoms (58%), and injuries due to severe weather (57%). A majority reported that certain specific groups of people will be disproportionately affected by climate change, including people with chronic diseases (75%), the poor and working poor (65%), young children aged 0 to 4 years (66%), and adults over age 60 years (63%). A large majority support education on climate change and health in the form of continuing medical education (74%), undergraduate medical education (73%), patient education materials (71%), and association policy statements (77%), and a large majority agreed that physicians and their associations should be involved in advocacy pertaining to the health effects of climate change (75%).

In 2015, in an email survey to U.S. members of the American Academy of Allergy Asthma and Immunology (AAAAI) (n=1,184; response rate=22%), Sarfaty and colleagues(11) found that a large majority of respondents think that human-caused climate change is happening (81%), and that it is driven by human activity (76%), although only a minority felt “very” (10%) or “moderately” (33%) knowledgeable about climate change and health. A majority (83%) indicated that climate change is relevant to direct patient care (83%), and reported that climate change is already affecting the health of their own patients (72%); the most common health effects identified were: air pollution-related increases in severity of chronic disease, such as asthma, COPD, pneumonia, cardiovascular disease (73%); increased care for allergic sensitization and symptoms of exposure to plants or mold (visits to office/ER for asthma/ allergic symptoms) (63%); and injuries due to severe storms, droughts, and fires (49%). Most respondents indicated that certain specific groups of people will be disproportionately affected by climate change, including people with chronic diseases (73%), young children aged 0-4 (57%), adults above age 60 (53%), and the poor and working poor (50%). A majority showed support for education on climate and health in the form of continuing medical education (71%), undergraduate medical education (67%), and patient education materials (66%). A majority also supported medical advocacy roles, including: Physicians should have a significant advocacy role (65%); Physicians have a responsibility to bring the health effects of climate change to the attention of the public (56%); AAAAI should have a significant advocacy role in relation to climate and health (61%); and AAAAI should have policies on climate change and health (67%).

*Other Clinicians*

In 2012, in an email survey conducted with dieticians who were members of the Academy of Nutrition and Dietetics (n=570, randomly sampled; response rate=15%), Hawkins and colleagues(12) found that most respondents (75%) indicated that climate change is an important issue, although slightly fewer than half felt it was an important practice issue for dieticians (45%). Over one third of respondents (38%) reported conducting activities that promote diet as a climate change mitigation strategy.

Non-English speaking nations

*Europe*

In 2012, in a survey of infectious disease experts responsible for climate change activities across 29 European Economic Area countries, plus Norway and Iceland, Semenza and colleagues found that found that a large majority agreed that climate change would affect vector-borne (86% of country representatives), food-borne (70%), and rodent-borne (69%) diseases. Respondents also indicated that improvements in institutional capacities for managing climate vulnerabilities are needed, including surveillance programs (83%), collaboration with the veterinary sector (69%), management of animal disease outbreaks (66%), national monitoring and control of climate-sensitive infectious diseases (64%), health services during and infectious disease outbreak (61%), and diagnostic support during an epidemic (54%).(13)

*Africa*

In 2011, in a survey of all students (medicine, environmental health, nursing, pharmacy, public health, etc.) in the College of Health Science of Haramaya University, Ethiopia, Nigatu and colleagues(14) found that over three quarters of the students were aware of health consequences of climate change, ranging from 61% among pharmacy students to 100% among environmental health and post-graduate public health students, but almost all (88%) students stated that their knowledge was insufficient to fully understand the public health impacts of climate change. Students who were more knowledgeable about climate change were dramatically more likely to perceive it as a serious health threat than those who were less knowledgeable [OR: 17.8, 95% CI: 8.8-32.1].

*Asia*

In 2008, in a paper & pencil survey of medical interns in five medical colleges in a coastal town in Karnataka, India (n=130; response rate=20%), Majra and Acharya(15) found that all respondents were aware that human-caused was occurring. Large majorities were aware than climate change can have health impacts including direct harm from extreme climatic events (945), increases in vector-borne diseases (78%), population displacement (78%), malnutrition (78%) and water-borne diseases (75%). Prevention response options including reducing fossil fuel use (92%) and education and communication (95%) were mentioned by nearly all responds; conversely, few mentioned adaptation (7%) and disease surveillance (6%) as strategies.

In 2013, in a survey of participants in three stakeholder workshops (consisting mostly of health professionals) in Phnom Penh, Siem Reap, and Kratie, Cambodia (n=66), McIver and colleagues(16) found that understanding of the health risks posed by climate change is low; most respondents (85%) indicated that they knew “a little” about the impacts of climate change on human health, with the remainder (15%) indicating that they knew “a lot.” In response to an open-ended question, the large majority of respondents (85%) reported at least one accurate health problem – most commonly diarrheal disease (74%) and acute respiratory disease (32%) – and many identified unrelated conditions.

In 2013, using a paper & pencil survey of staff in selected departments (disease control, public health, medical laboratory, emergency response) of the Centers for Disease Control & Prevention in Shanxi Province (n=314; response rate=95%), Wei and colleagues(17) found that a majority feel that global (66%) or regional (69%) climate change is occurring, and most also believe that human activities are the main cause of climate change (74%). Nearly all respondents agreed with the statement that climate change would affect human health (87%), with more than half (57%) indicating the health impacts will be exclusively negative, and many others (40%) indicating both negative and positive health impacts. Large majorities of respondents strongly supported mitigation actions by the central government (optimizing the industrial structure and saving energy – 87%; developing low-carbon energy – 85%; controlling emission of GHGs – 74%) but fewer (30% to 55%) supported various health adaptation actions, or strengthening in-house training.

In 2014, using a paper & pencil survey with randomly selected nurses from six public hospitals in central China (n=293; response rate=89%) Xiao and colleagues(18) found that most respondents (76%) felt that climate change would affect public health, although only 33% felt it would affect their work. A large majority of respondents felt it was necessary to learn more about climate change and health (89%).

In 2015, using a paper & pencil survey of staff in selected departments (infectious disease control, public health, medical laboratory, emergency response) of the Centers for Disease Control & Prevention in Guangdong Province (n=260; response rate=93%), Tong and colleagues(19) found that the majority of respondents (75%) were concerned about climate change, and believed that climate change would have a negative effect on population health (81%). However, only a minority (27%) reported having a good understanding of climate change, and the large majority (85%) indicated they needed more information about the health impacts of climate change.

**RQ2: How does the public perceive the health impacts of climate change?**

English speaking nations

In approximately 2006 (actual data collection dates not reported), an interviewer administered survey of residents of St. Kitts & Nevis and Trinidad & Tobago (n=227 in SKV and n=650 in TT, cluster sampled) found that a only small minority of island residents (7% in SKN and 14% in TT) specifically identified harm to health as an impact of climate change, although many more (55% and 45%, respectively) answered “all of the above” to the question – which included health impacts.(20) When asked to specify how health on the islands was harmed by climate change, most respondents could not identify a specific form of health harm (57% and 54%,); of those who did, the most common health impacts identified were heat stress (10% and 11%), food-borne disease (7% and 18%), water-borne disease (7% and 16%), respiratory disease (9% and 4%).

In 2007, in-depth interviews were conducted with older (ages 72 to 94) British adults (n=77; convenience sample) in London and Norwich who live in their own homes.(21) Respondents tended not to see themselves at risk of health problems during heat waves, nor were they aware that their pre-existing medical conditions placed them at higher risk. Some recognized that older people are at risk, but they did not feel at risk themselves. Most were able to describe appropriate actions to take during a heat wave.

In 2008, a telephone survey of American adults (n=771, randomly sample) showed that – in response to closed-ended questions asking if “climate change causes the following types of environmental impacts in the United States” – many respondents answered that it contributes to the spread of infectious diseases (55%), reduced food production (55%), and increased pollen (44%).(22)

In 2008, a web-based survey of American adults (n=2,164, randomly sampled) found that no respondent answered an open-ended question about “the first thing that comes to mind when you hear the term global warming” with a response pertaining to human health.(23,24) Yet, in response to specific questions about when global warming will “start to harm” people in the U.S. and other people around the world (with no specific reference made to health), 34% responded American are being harmed “now” and 38% responded people elsewhere are being harmed now. Respondents had a strong tendency to see people distal to them as being at greater risk for harm from global warming (future generations: 61%; people in developing countries: 53%; people in the U.S.: 49%) rather than people close to them (people in your community: 39%; your family: 35%; you personally: 32%). When asked to estimate the number of people worldwide who currently become injured or ill each year worldwide from global warming, 5% estimated “millions,” 15% “thousands,” 13% “hundreds,” 21% “none,” and 46% said “don’t know.” When asked to estimate the number of people worldwide who currently die each year worldwide from global warming, 3% estimated “millions,” 14% “thousands,” 12% “hundreds,” 23% “none,” and 48% said “don’t know.”

In 2008, a telephone survey of Canadian adults (n=1,600, randomly sampled) showed that few Canadians made the connection between climate change and health harms unprompted, but when asked specifically, a large majority give answers that indicate they see climate change as having considerable potential for health harm).(24,25) Specifically, when asked an open-ended question about the environmental problems that pose the greatest risk to their nation’s public health, only 10% mentioned climate change, while 54% mentioned air pollution or smog. However, in response to an open-ended question about how climate change can harm the health of respondents, 60% did name at least one health harm, with the most common being respiratory problems (22%), infectious diseases (11%), cancer (11% - which is not a major health impact of climate change), and air quality problems (8%). And in response to an open-ended question about Canadians most likely “to experience the negative effects of climate change,” many respondents were able to name the elderly (45%), children (33%), and people with illnesses (14%). When asked specifically if they believed that climate change would increase the risk of various health problems, large majorities indicated in the affirmative for respiratory problems (78%), heat stroke (75%), injuries from extreme weather events (73%), and infection diseases (62%). Fully two thirds (67%) said they are personally vulnerable to the potential health impacts of climate change, and over three quarters (76%) said that people in their community are vulnerable.

In 2009, a face-to-face survey of Inuit elders and seniors (n=75), and an in-depth follow-up interview (n=22) in Rigolet, Nunatsiavut, Canada found that 64% of respondents felt that climate change was having an impact on their health.(26) The primary health impacts reported were reduced physical activity (due to a shorter ice season), reduced nutrition (due to reduced catch of wild game), and increased stress and substance use.

In 2010, in-depth interviews (n=22, convenience sample) with adults in Ontario (Canada) showed that residents had little understanding of the ways in which climate change influences health.(27)

In 2012, most (80%) adults in Adelaide (AU) report being concerned about heat waves, but relatively few (27.5%) were concerned about their health.(28) Main reasons for concern were personal comfort (61%), the garden (49%), and sleeping well (48%).

In 2013, in a mail survey with adult residents of Maryland (n=2,126, randomly sampled), more than half of respondents felt that climate change posed a moderate or large risk to people’s health in general (54%), and to their own health (56%).(29)

In 2013, a web-based survey of American adults (n=1,321, randomly sampled) found that on average, Americans have a strong tendency to see climate change as less threatening to their and their family’s health (4.8 on a 0 to 10 scale) to than to other people’s health – with escalating levels of health threat seen for people in their community (5.0), Americans in general (5.9), and people worldwide (6.6).(30)

In 2014, a web-based survey of American adults (n=1,275, randomly sampled) showed that relatively few Americans make the connection between climate and health unprompted, but when asked specifically, a majority indicated that climate change is “bad” for people’s health.(31) Specifically, relatively few people (32%) said they had given thought to how global warming might affect people’s health. In response to a closed-ended affective response question, most people (64%) indicated GW is “bad” for health, but in response to a subsequent open-ended question, few people (27%) could name even one specific type of harm (with respiratory problems being most commonly category mentioned, at 11%, and extreme weather/natural disasters as the second most commonly mentioned impact at 6%). Moreover, while 33% indicated that some groups are more affected than others, only 25% could name any specific group (the most commonly mentioned group was seniors (8%), followed by the poor/homeless and people with chronic conditions (7% each)). In response to closed-ended question, a majority (57%) felt the health of Americans was being harmed at least “a little” by global warming, while large minorities felt the health of people in their household (40%), and their own health (42%) were being harmed at least “a little.” The health conditions that people felt would become more common over the next decade, if nothing is done about global warming, were air pollution (38%), allergies (38%), asthma (37%), heat stroke (36%), bodily harm from severe storms (34%), diseases carried by insects (34%); illnesses from food- or water-borne bacteria and viruses (32%), hunger/malnutrition due to rising food prices (30%), bodily harm from flooding (27%), severe anxiety (27%), bodily harm from wildfires including smoke inhalation (26%), and depression (26%), but it must be noted that many respondents also felt that cancer (31%) and influenza (29%) would increase over the next decade (if nothing is done about global warming), therefore these latter findings must be considered with all due caution.

In 2016, a web-based survey of American adults (n=1,226, randomly sampled) found that, in response to a closed-ended question, 62% said that global warming is a health issue. This was comparable to the proportion that said it was an economic issue (60%), a severe weather issue (61%), and an agricultural (food/farming) issue (65%), and less than those who said is was an environment issue (76%) or a scientific issue (70%).(23)

Non-English speaking nations

*Africa*

In 2011, a face-to-face survey of Tanzanian heads of household in land-locked Kilosa District (n=390, based on a random sample) found that while few respondents knew the term climate change (25%), nearly all respondents (95%) felt the rain patterns had changed over the past 10 years, and over half (62%) felt it had gotten warmer during that period.(32) Food insecurity was reported to be endemic, with 73% reporting household food shortages in the past five years. Food shortages were most likely to be reported during the rainy season, when most households experience febrile illnesses including malaria.

In approximately 2012 (data collection dates not provided), a face-to-face survey of rural southwestern Nigerian adults (n=1,019; randomly sampled) found that nearly all respondents (89%) felt their climate had been changing over the past 10 years – with changes in precipitation patterns being the primary manifestation – although most believed the causes to be supernatural (e.g., God, human sins; 49%) or natural (30%), while a small minority believed the changes to be human-caused (15%).(33) A large majority of respondents felt the changing climate was having negative impacts on adults (illness, 46%; stress/suffering, 25%, hunger, 12%), on children (illness, 70%; hunger/malnutrition, 7%; poor growth, 8%), on food production (reduced production, 80%; increased cost, 39%), and on water supplies (wells/rivers drying up, 61%; reduced supplies, 56%).

*Asia*

In 2010 and 2011, a survey of adults in two Bangladeshi villages (n=450, randomly sampled) showed that nearly all respondents (95%) felt that summers had gotten hotter in recent years, and a large majority (80%) felt that health problems had increased as a result.(34)

In 2012, a face-to-face survey of adults in seven vulnerable districts in Bangladesh (n=6,720, randomly sampled) showed that while only about half of respondents (54%) had heard of climate change, a large majority felt that, over the past decade, rainfall patterns had changed (92%), drought had increased (85%), food production had decreased (71%), and health care expenditures had increased as a result of increasing extreme weather events.(35)

In 2012, a face-to-face survey of adult residents of four districts in Lhasa City, Tibet (n=619, randomly sampled) found that nearly all respondents believed the temperatures in Lhasa had been increasing over the past few decades (90%) and that the rising temperatures are a threat to health, directly or indirectly (92%). Most (78%) also believed the rising temperatures are a threat to their health.(36)

In 2012 and 2013, a face-to-face survey of adult residents in both slum and non-slum areas of Hanoi, Vietnam (n=1,412, randomly selected) found that most participants (79% in non-slum areas and 70% in slum areas) had heard of climate change – with approximately two-thirds of participants associating it with more storms, more floods, and longer heat waves. Nearly all respondents (91% in non-slum areas and 92% in slum areas) indicated that climate change harms health – which was the commonly perceived impact.(37) Over one third of respondents in both areas felt their families were sick more frequently during the past summer (35%, 38%), and during the past winter (44%, 37%), in comparison to five years ago, while few (2 to 3%) felt their families had been sick less frequently.

In 2013 and 2014, a face-to-face survey of adults in Bhimad village, Nepal (n=258, randomly sampled) showed that about half (55%) felt their climate had change, that it is getting hotter in summer (54%), and rainier during the rainy season (49%), and there has been an increase in disease during the summer (50%), winter (49%) and rainy season (46%) – although participants were not asked if they felt the increase in disease was attributable to the change in climate.(38)

*Europe (Malta)*

In 2009, a telephone survey of Maltese adults (n=543, randomly selected) found that a large proportion of Maltese felt that climate change can cause illness (89%) and death (77%), with many saying that it already is (illness: 63%; deaths: 50%) (Akerlof et al., 2010). When given a list of health problems that are potentially caused by climate change, most respondents identified asthma and respiratory difficulties (91%), skin cancer (90%), heat wave events (84%), and allergies (84%) as being associated with climate change; conversely, only about a third identified cardiovascular problems, and just under half identified infectious and diarrheal diseases. Note: All of these findings were from closed-ended questions.

**RQ3. How does the public react to information about the health implications of climate change?**

In 2017, a face-to-face survey of students at the University of Exeter (n=240, based on a random sample) found that while frames prioritizing public health led to significantly higher support for policies to reduce cars use in comparison with climate change prioritizing frames, but that the relative effectiveness of public health and climate change framings depended on participants’ perception that a policy would produce health benefits, i.e., its relevance.(39)

In 2010, semi-structured in-depth elicitation interviews with a demographically and geographically diverse group of American adults (distributed equally across the "Global Warming’s Six Americas" audience segments; n=74) showed that a brief (approximately 400 word) essay about the human health relevance of climate change appeared to have provided participants across the six audience segments with a useful and engaging new frame of reference for climate change. Across all six audiences, the most compelling information pertained to the health benefits of responding to climate change (rather than the health risks caused by climate change).(40)

In 2011, in a web-based randomized controlled trial with American adults (N=1,127, randomly sampled), participants were shown one of three fabricated news articles making the case for the climate change impacts on (and climate response benefits to) one of three issue frames: human health, national security, the environment. The public health article was the most likely to elicit emotional reactions consistent with support for climate change mitigation and adaptation.(41)

In 2012, in a randomized controlled trial testing the effect of information about the health impacts of climate change on outdoor workers either nearby (upstate New York) or far away (the state of Georgia or the country of France), information about both groups of affected workers increased support of climate change mitigation policies among Democratic participants (as compared to a control message), but reduced support among Republic participants, especially in the “high social distance” (i.e., far away) condition.(42)

In 2013 and 2014, equivalent surveys were conducted in 24 countries with university students (N=4,449, convenience sampled) and in 10 countries with community samples (N=1,239, convenience sampled) all inhabited continents, using the local language for each country. Participants were not presented with information, but rather were asked to imagine what their country will look like in the future – in 2050 – when people have taken action aimed to preventing climate change. They were then asked how much better, or worse, various conditions in their society would be at that time, including disease and pollution. Participants who believed that health benefits will occur as a result of taking national action against climate change were significantly (albeit weakly) more likely to intend to perform climate change relevant citizenship actions (e.g., contact an elected official) and household actions (e.g., install products to save energy) over the next 12 months, and to donate to an environmental organization.(43)

In 2014, in a web-based randomized controlled experiment with American adults (N=1,591, convenience sample), participants were shown one of four fabricated news articles making the case for the benefits of climate change based on one of four issue frames (economic opportunity, national security, Christian stewardship, and public health); half of the participants in each condition were also shown a climate change denial counter-message. The public health framed news article positively influenced participant’s beliefs that policies to reduce America’s greenhouse gas emissions will benefit American’s health; the effect was eliminated, however, when the news article was accompanied by the climate change denial message. (Note: The news articles framed on economic opportunity and national security were effective even when accompanied by the climate denial message.)(44)

In 2015, in a randomized controlled trial of the effect of information (environmental and public health impacts of electricity production versus monetary saving) on household energy consumption (N=118 households), information about avoidable environmental and public health impacts (e.g., pounds of pollutants, childhood asthma, and cancer) outperformed information about monetary savings over the 100-day experimental period. Overall, participants who received messages emphasizing air pollution and health impacts associated with energy use reduced their consumption by 8.2% (and families with children reduced significantly more – 19%), while participants in the cost savings information group showed no significant reduction.(45)

In 2015, in a randomized, controlled web-based experiment with American adults (N=672, convenience sample), participants read one of two first-person brief essays (approximately 200 words) emphasizing either the risks of failing to combat climate change or how combatting climate change will improve health through pollution reduction and encouraging more active lifestyles. Reactions to the two essays on three sets of outcome measures – policy support, beliefs about the impacts associated with personal behavior change, and environmental advocacy intentions – were equivalent, and the authors concluded that “simple reframing (is) unlikely to boost public support for climate policy.”(46)

In 2016, in a web-based experiment with American adults (N = 207 Facebook users, convenience sampled), participants were shown 10 climate change co-benefit messages (2 each of five types, including health messages), and asked to pass them on (in their own words, from memory) to the Facebook friends. Those messages, in turn, where shown to a second group of participants who were asked to do that same (pass them on, in their own words, from memory to their Facebook friends). The human health (and nature) messages were more likely to survive the chain of interpersonal transmission (i.e., were more likely to reach the end of the person-to-person-to-person transmission) than messages about societal competence (e.g., applying scientific knowledge), communality (e.g., being more community-minded), and economic development (e.g., helping the economy).(47)

In 2016, in a web-based study with American adults who were vulnerable to the health effects of climate change (having chronic conditions and low-SES; N=122), participants were shown materials that explained (using graphics and concise, simple language) how climate change affects health conditions and how to engage in protective adaptation behaviors. In response, participants become more certain that climate change is happening, and that climate change is affected their health, and they gained knowledge about who is most vulnerable to extreme heat, and environmental conditions that increase allergy-producing pollen. Participants were most interested in advice on health management and protective behaviors related to their chronic conditions, and less interested in messages about collective action to slow or stop climate change. Post-hoc analysis showed that knowledge about climate and health, and believing that climate change is affecting their (or household member’s) health were both positively associated with intentions to engage in climate change adaptation and mitigation actions.(48)

In 2016, in a field experiment conducted in a five American states (two in the northeast, one in the mid-Atlantic, and two in the west), women over the age of 25 (whose email addresses were acquired from a liberal-leaning organization that describes itself as the “world’s largest online community for good”) were sent one of two email messages: a control messages that briefly “described the problem of climate change and then quickly transitioned to efficacy-boosting language about what we have to gain from climate change mitigation policies” (N=51,322); and health message that included the control text in entirety but also added a short passage in the middle prompting readers to think about how climate change would threaten their personal health (N=51,234). All message recipients were asked to sign a petition in support of climate action; participants who received the health message signed the petition at a rate 13% below that of participants who received the control message – thus the health message significantly reduced the rate of the requested behavior. In a second study, people in a separate online panel (similar in composition to the sample used in the field study; N=645) received either the control message or the health message and were asked a series of questions to assess their perceptions about the personal relevance of climate change, and their support for climate mitigation policies. In this experiment, recipients of the health message showed significantly greater concern about climate change, and greater support for climate action. In a final study, using similar methods to the second study, control condition and health message condition participants were asked if they would like to join a prominent climate advocacy organization; 11% fewer participants who received the health message signed up. The authors of these studies conclude – consistent with their hypothesis – that climate messages that make people concerned for their own health heighten people’s concerns about climate change, but reduce their rate of political participation to express that concern.(49)

**REFERENCES**

1. Maibach EW, Chadwick A, McBride D, Chuk M, Ebi KL, Balbus J. Climate Change and Local Public Health in the United States: Preparedness, Programs and Perceptions of Local Public Health Department Directors. PLOS ONE. 2008 Jul 30;3(7):e2838.

2. Bedsworth L. Preparing for Climate Change: A Perspective from Local Public Health Officers in California. Environ Health Perspect Res Triangle Park. 2009 Apr;117(4):617–23.

3. Carr JL, Sheffield PE, Kinney PL. Local Preparedness for Climate Change among Local Health Department Officials in New York State: A Comparison with National Survey Results. J Public Health Manag Pract JPHMP. 2012;18(2):E24–32.

4. Syal SS, Wilson RS, Crawford JM, Lutz J. Climate change and human health—what influences the adoption of adaptation programming in the United States public health system? Mitig Adapt Strateg Glob Change. 2011 Dec 1;16(8):911–24.

5. Paterson JA, Ford JD, Ford LB, Lesnikowski A, Berry P, Henderson J, et al. Adaptation to climate change in the Ontario public health sector. BMC Public Health. 2012;12:452.

6. Polivka BJ, Chaudry RV, Mac Crawford J. Public Health Nurses’ Knowledge and Attitudes Regarding Climate Change. Environ Health Perspect Res Triangle Park. 2012 Mar;120(3):321–5.

7. Roser-Renouf C, Maibach EW, Li J. Adapting to the Changing Climate: An Assessment of Local Health Department Preparations for Climate Change-Related Health Threats, 2008-2012. PLOS ONE. 2016 Mar 18;11(3):e0151558.

8. Purcell R, McGirr J. Preparing rural general practitioners and health services for climate change and extreme weather. Aust J Rural Health. 2014 Feb 1;22(1):8–14.

9. Sarfaty M, Mitchell M, Bloodhart B, Maibach EW. A Survey of African American Physicians on the Health Effects of Climate Change. Int J Environ Res Public Health. 2014 Nov 28;11(12):12473–85.

10. Sarfaty M, Bloodhart B, Ewart G, Thurston GD, Balmes JR, Guidotti TL, et al. American Thoracic Society member survey on climate change and health. Ann Am Thorac Soc. 2015 Feb;12(2):274–8.

11. Sarfaty M, Kreslake JM, Casale TB, Maibach EW. Views of AAAAI members on climate change and health. J Allergy Clin Immunol Pract Amst. 2016 Mar;4(2):333–5.

12. Hawkins IW, Balsam AL, Goldman R. A Survey of Registered Dietitians’ Concern and Actions Regarding Climate Change in the United States. Front Nutr [Internet]. 2015 Jul 8 [cited 2017 Mar 23];2. Available from: http://www.ncbi.nlm.nih.gov/pmc/articles/PMC4495332/

13. Semenza JC, Suk JE, Estevez V, Ebi KL, Lindgren E. Mapping climate change vulnerabilities to infectious diseases in Europe. Environ Health Perspect. 2012 Mar;120(3):385–92.

14. Nigatu AS, Asamoah BO, Kloos H. Knowledge and perceptions about the health impact of climate change among health sciences students in Ethiopia: a cross-sectional study. BMC Public Health. 2014;14:587.

15. Majra J, Acharya D. Protecting health from climate change: Preparedness of medical interns. Indian J Community Med. 2009;34(4):317.

16. McIver LJ, Chan VS, Bowen KJ, Iddings SN, Hero K, Raingsey PP. Review of Climate Change and Water-Related Diseases in Cambodia and Findings From Stakeholder Knowledge Assessments. Asia Pac J Public Health. 2016 Mar 1;28(2_suppl):49S–58S.

17. Wei J, Hansen A, Zhang Y, Li H, Liu Q, Sun Y, et al. Perception, attitude and behavior in relation to climate change: A survey among CDC health professionals in Shanxi province, China. Environ Res. 2014 Oct;134:301–8.

18. Xiao J, Fan W, Deng Y, Li S, Yan P. Nurses’ knowledge and attitudes regarding potential impacts of climate change on public health in central of China. Int J Nurs Sci. 2016 Jun;3(2):158–61.

19. Tong MX, Hansen A, Hanson-Easey S, Xiang J, Cameron S, Liu Q, et al. Perceptions of capacity for infectious disease control and prevention to meet the challenges of dengue fever in the face of climate change: A survey among CDC staff in Guangdong Province, China. Environ Res. 2016 Jul;148:295–302.

20. Rawlins SC, Chen A, Rawlins JM, Chadee DD, Legall G. A knowledge, attitude and practices study of the issues of climate change/variability impacts and public health in Trinidad and Tobago, and St Kitts and Nevis. West Indian Med J. 2007;56(2):115–121.

21. Abrahamson V, Wolf J, Lorenzoni I, Fenn B, Kovats S, Wilkinson P, et al. Perceptions of heatwave risks to health: interview-based study of older people in London and Norwich, UK. J Public Health. 2009 Mar 1;31(1):119–26.

22. Semenza JC, Ploubidis GB, George LA. Climate change and climate variability: personal motivation for adaptation and mitigation. Environ Health. 2011;10:46.

23. Leiserowitz A, Maibach E, Roser-Renouf C, Rosenthal S, Cutler S. Climate change in the American mind: November 2016. Yale University and George Mason University. New Haven, CT: Yale Program on Climate Change Communication; 2017.

24. Akerlof K, DeBono R, Berry P, Leiserowitz A, Roser-Renouf C, Clarke K-L, et al. Public Perceptions of Climate Change as a Human Health Risk: Surveys of the United States, Canada and Malta. Int J Environ Res Public Health. 2010 Jun 14;7(6):2559–606.

25. Berry P, Clarke K, Pajot M, Hutton D, Verret M. The role of risk perception and health communication in adapting to the health impacts of climate change in Canada. Nat Resour Can [Internet]. 2009 [cited 2017 Mar 24]; Available from: http://www.climateaccess.org/sites/default/files/Berry_The%20Role%20of%20Risk%20Perception%20and%20Health%20Communication.pdf

26. Ostapchuk J, Harper S, Willox AC, Edge VL, Government RIC. Exploring Elders’ and Seniors’ Perceptions of How Climate Change is Impacting Health and Well-being in Rigolet, Nunatsiavut. Int J Indig Health. 2012;9(2):6–24.

27. Cardwell FS, Elliott SJ. Making the links: do we connect climate change with health? A qualitative case study from Canada. BMC Public Health. 2013;13:208.

28. Akompab DA, Peng Bi, Williams S, Saniotis A, Walker IA, Augoustinos M. Climate Change, Community Understanding and Emotional Responses to the Impacts of Heat Waves in Adelaide. Int J Clim Change Impacts Responses. 2013 Apr;4(2):109–26.

29. Akerlof KL, Delamater PL, Boules CR, Upperman CR, Mitchell CS. Vulnerable Populations Perceive Their Health as at Risk from Climate Change. Int J Environ Res Public Health. 2015 Dec 4;12(12):15419–33.

30. Stoutenborough JW, Vedlitz A, Xing X. Are all risk perceptions created equal? Comparing general risk assessments and specific risk assessments associated with climate change. Hum Ecol Risk Assess Int J. 2016 Jan 2;22(1):50–70.

31. Maibach EW, Kreslake JM, Roser-Renouf C, Rosenthal S, Feinberg G, Leiserowitz AA. Do Americans Understand That Global Warming Is Harmful to Human Health? Evidence From a National Survey. Ann Glob Health. 2015 May;81(3):396–409.

32. Mayala BK, Fahey CA, Wei D, Zinga MM, Bwana VM, Mlacha T, et al. Knowledge, perception and practices about malaria, climate change, livelihoods and food security among rural communities of central Tanzania. Infect Dis Poverty. 2015;4:21.

33. Asekun-Olarinmoye E, Bamidele JO, Odu OO, Olugbenga-Bello AI, Abodunrin OL, Adebimpe WO, et al. Public perception of climate change and its impact on health and environment in rural southwestern Nigeria [Internet]. Research and Reports in Tropical Medicine. 2014 [cited 2017 Mar 23]. Available from: https://www.dovepress.com/public-perception-of-climate-change-and-its-impact-on-health-and-envir-peer-reviewed-fulltext-article-RRTM

34. Haque MA, Yamamoto SS, Malik AA, Sauerborn R. Households’ perception of climate change and human health risks: A community perspective. Environ Health. 2012;11:1.

35. Kabir MI, Rahman MB, Smith W, Lusha MAF, Azim S, Milton AH. Knowledge and perception about climate change and human health: findings from a baseline survey among vulnerable communities in Bangladesh. BMC Public Health. 2016;16:266.

36. Bai L, Cirendunzhu, Pengcuociren, Dawa, Woodward A, Liu X, et al. Rapid warming in Tibet, China: public perception, response and coping resources in urban Lhasa. Environ Health. 2013;12:71.

37. Rocklöv J, Bao Giang K, Van Minh H, Ebi K, Nilsson M, Sahlen K-G, et al. Special Issue: Climate Change and Health in Vietnam. Glob Health Action [Internet]. 2014 Dec 8 [cited 2017 Mar 24];7. Available from: http://www.ncbi.nlm.nih.gov/pmc/articles/PMC4265652/

38. Mishra SR, Bhandari PM, Issa R, Neupane D, Gurung S, Khanal V. Climate change and adverse health events: community perceptions from the Tanahu district of Nepal. Environ Res Lett. 2015;10(3):034007.

39. Walker BJA, Kurz T, Russel D. Towards an understanding of when non-climate frames can generate public support for climate change policy. Environ Behav. 2017 Jul 7;0013916517713299.

40. Maibach EW, Nisbet M, Baldwin P, Akerlof K, Diao G. Reframing climate change as a public health issue: an exploratory study of public reactions. BMC Public Health. 2010;10:299.

41. Myers TA, Nisbet MC, Maibach EW, Leiserowitz AA. A public health frame arouses hopeful emotions about climate change. Clim Change. 2012 Aug 1;113(3–4):1105–12.

42. Hart PS, Nisbet EC. Boomerang Effects in Science Communication: How Motivated Reasoning and Identity Cues Amplify Opinion Polarization About Climate Mitigation Policies. Commun Res. 2012 Dec 1;39(6):701–23.

43. Bain PG, Milfont TL, Kashima Y, Bilewicz M, Doron G, Garðarsdóttir RB, et al. Co-benefits of addressing climate change can motivate action around the world. Nat Clim Change. 2016 Feb;6(2):154–7.

44. McCright AM, Charters M, Dentzman K, Dietz T. Examining the Effectiveness of Climate Change Frames in the Face of a Climate Change Denial Counter-Frame. Top Cogn Sci. 2016 Jan 1;8(1):76–97.

45. Asensio OI, Delmas MA. Nonprice incentives and energy conservation. Proc Natl Acad Sci. 2015 Feb 10;112(6):E510–5.

46. Bernauer T, McGrath LF. Simple reframing unlikely to boost public support for climate policy. Nat Clim Change. 2016 Jul;6(7):680–3.

47. Connor P, Harris E, Guy S, Fernando J, Shank DB, Kurz T, et al. Interpersonal communication about climate change: how messages change when communicated through simulated online social networks. Clim Change. 2016 Jun 1;136(3–4):463–76.

48. Kreslake JM, Price KM, Sarfaty M. Developing effective communication materials on the health effects of climate change for vulnerable groups: a mixed methods study. BMC Public Health. 2016;16:946.

49. Levine A, Kline. A new approach for evaluating climate change communication. Clim Change Lett. 2017 In press;

**Declaration of interests: We attest that we have no financial or personal relationships with other people or organizations that could inappropriately influence this work.**
